# Senior students’ experience as tutors of their junior peers in the hospital setting

**DOI:** 10.1186/s13104-015-1729-0

**Published:** 2015-12-02

**Authors:** Antonia J. Clarke, Annette Burgess, Audrey Menezes, Craig Mellis

**Affiliations:** Sydney Medical School-Central, The University of Sydney, Building 63, Level 4, Missenden Road, Camperdown, NSW 2050 Australia; Royal Prince Alfred Hospital, Missenden Road, Camperdown, NSW 2050 Australia; Hornsby Ku-Rin-Gai Hospital, Palmerston Road, Sydney, NSW Australia

**Keywords:** Education, Peer-assisted learning, Clinical skills, Medical students, Professionalism, Community

## Abstract

**Background:**

Student-led teaching has long been regarded as a useful ancillary educational method. It is also a valuable tool in the development of aspects of professionalism in student tutors and contributes to a sense of community within the student body. In 2014, a peer-assisted learning (PAL) program, organised by students at Sydney Medical School (Central), explored students’ experience of tutoring their junior peers.

**Methods:**

Year 3 and 4 students within Central Clinical School (CCS) were invited to be tutors for Year 1 and 2 students respectively. Tutorials centered on the application of clinical skills. All tutors were asked to complete an end of year questionnaire.

**Results:**

A total of 40 % of senior students participated as tutors and 65 % of junior students as tutees. The end of year questionnaire response rate was 48 % (20/42). Most tutors (19/20, 95 %) felt confident to teach tutorials although one-third (6/20, 30 %) would have preferred more training in teaching. Tutors felt that the program better prepared them for their exams. Almost all tutors (19/20, 95 %) enjoyed teaching and felt it fostered a sense of community at CCS (17/20, 85 %). Tutors stated they were likely to be involved in teaching in the future (17/20, 85 %).

**Conclusion:**

This student initiated PAL program provided tutors with the opportunity for content and clinical skills revision and assisted in the development of professional competencies required on entering the medical workforce. The resultant sense of community at CCS will aid the expansion of the program in 2015 with an aim to review quality assurance measures.

## Background

Peer assisted learning (PAL) is described as “people of similar social groupings who are not professional teachers helping each other to learn and learning themselves by teaching” [1]. PAL has long been regarded as a useful educational method [2]. The notion of PAL in the medical student context resulted from the informal exchange of shared interests within peer groups [1, 3]. More recently, research has focused on formalisation of peer-to-peer tutoring within medical programs [4, 5]. Peer-tutoring programs incorporate a wide range of medical skill sets, including case-based discussion [6], clinical examinations [2, 7], and student grand rounds [7]. Largely utilised as an ancillary teaching tool, PAL is designed to supplement and enhance students’ understanding of topics through interactive small-group activities [8].

The benefits of students acting as *peer tutors* have been widely reported, and include metacognitive gains, increased student responsibility, and development of professionalism skills [9, 10]. Since the 1990s there has been growing recognition of the importance of developing medical students’ teaching and assessment competencies, with peer teaching and assessment ability now increasingly documented internationally as a required graduate attribute of medical students [9, 11]. At Sydney Medical School—Central, senior students lead a peer tutoring program for junior students. We theorised that the experience as a peer tutor would provide our senior students with opportunities to revise medical knowledge and clinical skills, practice teaching, and contribute to the School’s social learning network.

### Context

This study took place at Central Clinical School (CCS), based at a large tertiary teaching hospital, and one of six clinical schools to which students are allocated during the 4-year graduate entry Sydney medical program (SMP). Year 1 and 2 students spend 1 day per week at the hospital. Year 3 and 4 are based at the hospital 5 days per week, and are also rotated offsite for subspecialty terms, including paediatrics, community medicine, perinatal and women’s health, and psychiatry.

Guided by existing PAL literature, a formal, vertically-integrated PAL program was organised at CCS with Year 3 and 4 students (the ‘tutors’) regularly teaching Year 1 and Year 2 students (the ‘tutees’) respectively. Although endorsed by CCS academic staff, the PAL program was designed to be student-driven and organised, and to supplement the existing CCS curricula. The main aims of the PAL program were threefold: to reinforce and extend curriculum competencies for the tutees; to develop tutoring skills and confidence amongst the tutors; and to enrich the sense of community within CCS.

This study aimed to explore students’ experience of tutoring their junior peers through the PAL program.

## Methods

The study took place over the course of the 2014 academic year.

All students within CCS (n = 207) were invited to take part in the PAL program. Participation was voluntary. All Year 3 students (n = 46) were invited to act as tutors for Year 1 students (n = 50), and Year 4 students (n = 60) as tutors for Year 2 students (n = 51).

### Program overview

Tutors were allocated in pairs, to ensure that at least one tutor was available for each tutorial. Tutors were allocated to the same group of three to four tutees for the entire year. Tutors were provided with a 1 h information session detailing the objectives and organisation of the program, and the format of the tutorials. It was suggested that tutorials be held fortnightly at a minimum, with the choice of more frequent sessions if desired.

### Tutorial content and format

Tutorials were approximately 1 h long, covering clinically relevant content. The topic of the tutorial was identified by tutees, who notified the tutors of this topic several days in advance of the tutorial, allowing them adequate time to prepare. Once the topic was identified, tutors largely drove the style and method of teaching and the depth of content. Tutorials were designed to supplement existing teaching in order to enhance the tutees’ knowledge base. The delivery was not intended to be didactic, but interactive, with the ideal tutorial having 20 min of theoretical content, 20 min of clinical application of the content through the examination of a patient on the wards or review of laboratory results, and 20 min for discussion of the case. The format was flexible according to tutor and tutee preference.

One-page handouts were created for content-driven tutorials. This encouraged tutors to remain concise and clear. Handouts were uploaded to a shared online folder, accessible by all participants in the program, allowing students to benefit from other tutorials as well as their own. In order to minimise the potential for teaching of incorrect content, three junior medical officers (JMOs) at the hospital with an interest in peer learning and prior involvement in similar programs kindly reviewed the content in the handouts in advance of the tutorials.

### Assessing tutors’ perceptions of the program

All participating tutors (n = 66) were asked to complete a questionnaire regarding their experience as a tutor. The questionnaire was designed to reflect the structural elements of Wegner’s ‘Communities of Practice’, and focused on joint enterprise (development of teaching skills), shared repertoire (academic and clinical knowledge) and mutual engagement (development of a sense of community) [12]. The questionnaire consisted of 12 closed items relating to their prior teaching experience and program outcomes. Responses ranged from “strongly disagree” (1) to “strongly agree” (5) on a five point Likert scale. The questionnaire also included open-ended questions aimed at eliciting responses from students regarding the most useful aspects of the program, and suggested improvements for the program.

Descriptive statistics were used to analyse the quantitative data [13]. Framework analysis was used to analyse qualitative data [13].

Ethics approval was obtained from The University of Sydney Human Research Ethics Committee.

## Results

### Participants

A total of 42/106 (40 %) students took part as tutors and 66/101 (65 %) students as tutees. Of the tutors, 19/46 (41 %) were Year 3 students and 23/60 (38 %) were Year 4 students. Two-thirds of tutors had completed the evidence based clinical teaching course ‘Teaching on the Run’ at CCS earlier in the year [14]. Of the tutees, 34/50 (68 %) were Year 1 students and 32/51 (63 %) Year 2 students.

### Frequency of tutor participation

Over the 7-month period of this program, the majority of tutors facilitated 4–6 tutorials, with the range from 1 to 13 tutorials.

### Survey responses

In total, 20/42 (48 %) of tutors completed the questionnaire. About half of these (n = 11) were Year 4 students, with 9 students in Year 3. Tutor responses were evenly split on gender. The median age of respondents was 26.

### Closed item responses

**Fig. 1:**
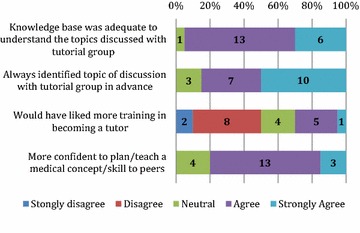
Tutor’s perception of preparation and development of teaching skills

**Fig. 2:**
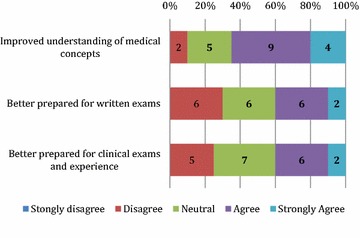
Tutor’s perception of benefits of participation in PAL program

**Fig. 3:**
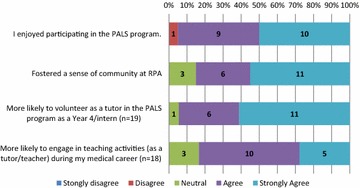
Tutor’s future teaching intentions and development of a sense of community

The responses are displayed in Figs. 1, 2, and 3 below. Students reported they were confident to tutor the arranged topic; that the program helped to develop their teaching ability; and better prepared them for their own exams. Only a minority (n = 60) of students felt they needed more training in teaching. Almost all students enjoyed the teaching experience and felt that the PAL program fostered a sense of community at CCS, with most students stated they were likely to be involved in teaching in the future.

### Free-text responses

Qualitative data consisted of open-ended question responses. These qualitative responses contextualised the questionnaire results. Analysis revealed four key themes, including tutors’ perceived: (1) development and revision of knowledge and clinical skills, (2) development and recognition of the importance of teaching skills, (3) development of community at CCS, and (4) suggested improvements to structure and administration of the program.

Students found the required tutor responsibilities of revising, preparing and teaching useful in reinforcing their own knowledge and clinical skills.

*‘It was useful for consolidating my own knowledge. I was required to deeply revise different topics.’*

*‘I always learn more when I teach.’*

Students perceived skills related to professionalism were developed through their teaching responsibilities, and recognised their importance to their future medical careers.

*‘It should be a requirement for all doctors to teach those more junior, it’s… an important part of transferal of knowledge.’*

*‘The chance to practice teaching….the sense of achievement [after each tutorial].’*

Students placed value on the professional experience gained from teaching, and found that participation fostered a desire for future involvement.

*‘I am eager to continue teaching as a JMO and later on in my own career.’*

Students found tutoring enjoyable and found the tutoring program built on the School community.

*‘I enjoy teaching, and this fostered a sense of community at Central Clinical School.’*

Students expressed altruistic reasons for tutoring, and valued the opportunity to be able to share their knowledge and contribute to the education of their junior peers.

*‘I was appreciative of past tutors and wanted to give back. I wanted to help the lower years in areas that I wish I’d had more help in…especially preparing for the OSCEs.’*

The drawbacks of the program were primarily logistical. Tutors wanted protected time for peer teaching, with *‘a formal time set aside for tutoring sessions to avoid timetabling issues.’* They also wanted more formalisation to the program, with a ‘*list of topics as suggestions’* and *‘a structured set program within the curriculum.’*

## Discussion

This study specifically sought to investigate tutors’ experience within the PAL program, particularly with regard to their confidence and preparedness for teaching and the associated benefits and challenges of peer-assisted learning. Tutors identified three key benefits of their participation, including knowledge and skills revision; development of professionalism attributes; and fostering a sense of community within CCS. Students also made suggestions to improve the program that largely related to timetabling and administration of the program.

### Tutors’ confidence and preparedness for teaching

The majority of tutors felt their knowledge base was adequate for each tutorial (19/20) and most students felt competent to teach. Two-thirds of tutors (28/42) had previously completed an evidence based clinical teaching course, ‘Teaching on the Run’, at CCS, reflecting a general interest by medical students in developing specific teaching skills [14, 15]. In addition, students at CCS are exposed to programs on receiving and providing peer feedback in the clinical setting from Year 1 [16]. Only 6/20 (30 %) of students desired formal training prior to becoming a tutor. It is unclear from the anonymity of tutor questionnaires whether those students who desired more training had previously completed the Teaching on the Run course, or indeed found the principles of the course applicable in the peer-to-peer setting. Future research should ensure standardisation of teaching skills in this context.

The student-driven nature of the PAL program meant that there was a strong reliance on self-directed preparation from the tutors. Actual tutor competence or performance was not assessed on an individual basis. Moreover, opportunities for corrective feedback regarding content were limited to JMO review of the handouts. Training in the health professions is increasingly exposed to unsupervised learning methodologies [15]. It is well established that students are capable of effectively modifying their cognition and motivation to achieve learning goals, however, these tools may be less effective when unsupervised [15]. The same may be the case for student tutors.

Self-assessed tutor competence should be interpreted with caution, with existing research suggesting that high performers generally under-estimate their own performance and to the contrary, lower performers tend to overestimate [17]. Importantly, however, feedback from student tutees was positive with 97/101 tutees stating the program improved their understanding of medical concepts and a large majority of tutees (91/101) felt that they were better prepared for their clinical exams and experience as a result of the PAL program. This finding is an important one and echoes other findings that tutees find their peers more approachable and communicate using a language similar to theirs [18–20].

### Benefits to tutor participation

#### Knowledge and skills revision

The development of the tutorial outline and content by tutors can facilitate deeper learning, and can help students to reflect and expand on their own knowledge [21]. Tutor feedback indicated that teaching of peers, particularly the preparation for the activity, allowed revision and reinforcement of their own knowledge and clinical skills. A large proportion of tutors (13/20) identified that the program developed their own understanding of medical concepts and felt better prepared for their own written and clinical exams (8/20).

#### Recognition and development of professionalism skills

Experience in peer teaching is an important tool in professional development [22]. It can foster high levels of responsibility in students, and self-reflection of teaching abilities [14]. Qualitative feedback indicated that tutors recognised teaching as a formal requirement in their future careers as medical practitioners. Survey responses indicated that as a result of participating as a tutor, students were more likely to engage in teaching activities in their future medical careers. Additionally, most tutors (16/20) felt more confident to plan and teach medical skills and concepts to their peers. Future research should focus on the development of meta-skills such as self-reflection and improvement [23, 24].

#### Development of a sense of community

Underscoring the program is the development of a sense of community within practice groups and across CCS. More than half (108/207) of the entire study body, from Year 1 to Year 4 had voluntarily chosen to take part in the program. Student responses highlighted altruistic reasons for volunteering to tutor their peers. Tutors felt that they were fostering a supportive environment for their junior peers. Most tutors (19/20) ‘*enjoyed*’ teaching their peers. Peer assisted learning relies on the construction of knowledge as a social attribute, rather than individually acquired knowledge [25]. Legitimate peripheral participation is a central notion to the development of a community of practice [26]. Through the PAL program, tutees and tutors are given the opportunity for meaningful participation within a community of practice. In particular, by engaging tutees as directors of tutorial content, the exchange of information through structured tutorials within the student community strengthened the sense of community at CCS and added to its social capital.

### Improving the PAL program

Tutors identified two key areas for improvement with regard to the planning and organisation of the PAL program. Students requested formal timetabling, with protected time for PAL teaching. A formal timetable may help to alleviate organisational requirements between tutors and tutees and therefore increase the frequency of tutorials to the suggested one tutorial per fortnight. Our data revealed that some tutors only held one tutorial, which was not expected. They also felt that a formal, ‘*structured set program*’ with identifiable topics within the curriculum would be beneficial in guiding tutorial topics. Whilst useful for the creation of learning goals, the use of a formal curriculum can be slow to adapt to changes in teaching methods and focus [25]. In future, a suggested list of PAL tutorial topics will be created from the SMP curricula and distributed to tutors at the beginning of the academic year. Timetabling, however, is more problematic, and will require consultation with CCS staff.

Future iterations of the program should focus on quality assurance in order to both assist tutors in development of their teaching skills, and provide quality assurance to the program. This could include the implementation of more intensive teacher training and assessment, with particular regard to the specific peer-to-peer learning environment, followed by direct observation, tutee evaluation, and provision of feedback on tutor performance by the organisers of the program.

Future research should also take into consideration the tutors prior exposure and experience to teaching skills, particularly in a post-graduate program such as the SMP.

## Limitations

The authors acknowledge that this is a local case study with a small sample size. Although the response rate was low, the findings were positive. In addition, the students who volunteered as tutors were self-selected, thus may be more likely to have a positive view of their experience [27].

## Conclusion

This study reviewed a student coordinated peer-to-peer tutoring program within Australia. The teaching and learning experience within the PAL program was highly regarded by students as tutors. It provided tutors with the opportunity for both content and clinical skills revision, better preparing students for their own exams. It also assisted in building students’ professional competencies required on entering the medical workforce. Pivotal to the development of this student-driven PAL program was the growing sense of community at CCS, which will aid the expansion of the program in 2015. While students appeared to have few concerns about the program, it is noted that implementation of a quality assurance process is lacking.

## Abbreviations

CCS: Central Clinical School
JMO: junior medical officer
PAL: peer assisted learning
SMP: Sydney medical program
Tutees: Year 1 and 2 students within SMP
Tutors: Year 3 and 4 students within SMP
